# Comparison of the Impact of Different Dacryocystorhinostomy Techniques on Patient Quality of Life in Lacrimal Duct Obstruction

**DOI:** 10.3390/jcm15124488

**Published:** 2026-06-10

**Authors:** Çağla Hakkani Öznay, Hüseyin Findik, Feyzahan Uzun, Muhammet Kaim, Murat Okutucu, Metin Çeliker, Mehmet Birinci

**Affiliations:** 1 Department of Ophthalmology, School of Medicine, Recep Tayyip Erdogan University, 53100 Rize, Turkey; caglahakkani94@gmail.com (Ç.H.Ö.); huseyin.findik@erdogan.edu.tr (H.F.); muhammet.kaim@erdogan.edu.tr (M.K.); murat.okutucu@erdogan.edu.tr (M.O.); 2 Department of Otorhinolaryngology, School of Medicine, Recep Tayyip Erdogan University, 53100 Rize, Turkey; meceliker@hotmail.com (M.Ç.); mehmet.birinci@erdogan.edu.tr (M.B.)

**Keywords:** dacryocystorhinostomy, epiphora, external dacryocystorhinostomy, endonasal dacryocystorhinostomy, nasolacrimal duct obstruction, transcanalicular multidiode laser, quality of life

## Abstract

**Objectives**: To prospectively compare the outcomes of external dacryocystorhinostomy (EX-DCR), endonasal dacryocystorhinostomy (EN-DCR), and laser-assisted transcanalicular dacryocystorhinostomy (TL-DCR) in patients with nasolacrimal duct obstruction (NLDO) and to evaluate the relationship between surgical outcomes and patient quality of life (QoL). **Methods**: This prospective comparative study included patients presenting with epiphora who were diagnosed with NLDO and scheduled for surgical treatment. Patients underwent EX-DCR, EN-DCR, or TL-DCR according to patient preference and nasal anatomical characteristics. All patients received bicanalicular silicone tube intubation. Follow-up examinations were performed on postoperative day 1, week 1, at month 1, and every three months thereafter. Anatomical success was defined as patency on nasolacrimal irrigation, and functional success was defined as a postoperative Munk score of ≤ 1 (complete or near-complete resolution of epiphora). QoL was assessed using the Lacrimal Symptom Questionnaire (Lac-Q) and the Glasgow Benefit Inventory (GBI). Statistical comparisons were performed between groups. **Results**: A total of 69 patients (45 women, 24 men; mean age 58.99 ± 14.86 years) were included, with a mean follow-up of 17.19 ± 4.07 months. The highest postoperative pain scores were observed in the EX-DCR group; with no significant intergroup differences. Anatomical success rates were 92% for EX-DCR, 90.5% for EN-DCR, and 91.3% for TL-DCR. Functional success rates were 88%, 90.5%, and 82.7%, respectively, with no statistically significant difference among techniques (*p* > 0.05). All groups showed postoperative improvement in Lac-Q and GBI scores. Numerically greater improvements were observed in the EN-DCR group, although intergroup differences were not statistically significant. **Conclusions**: EX-DCR achieved the numerically highest anatomical success rate, whereas EN-DCR demonstrated numerically greater improvement in patient-reported QoL outcomes. However, no statistically significant differences in anatomical success, functional success, or QoL measures were observed among the three techniques. Overall, all three procedures were effective surgical options for the treatment of NLDO.

## 1. Introduction

Epiphora secondary to primary acquired nasolacrimal duct obstruction (NLDO) is a frequent clinical problem that compromises visual function, ocular comfort, and social interaction in a meaningful proportion of patients. Although the condition is not life-threatening, chronic tearing, recurrent dacryocystitis, and the accompanying symptom burden can substantially impair patient-reported quality of life (QoL) [1].

When conservative measures fail, dacryocystorhinostomy (DCR) remains the standard surgical approach for restoring lacrimal drainage [2]. The external DCR (EX-DCR) technique, first described by Toti in 1904, has maintained its benchmark status through consistently high anatomical success rates [2]. Its requirement for a skin incision and disruption of medial canthal anatomy, however, carries cosmetic drawbacks and may prolong recovery. Endoscopic DCR (EN-DCR) has gained ground on the strength of its scarless, minimally invasive access to the lacrimal sac via the nasal cavity; in experienced hands, success rates approach those of EX-DCR [3]. Laser-assisted transcanalicular DCR (TL-DCR) has more recently entered practice as a further minimally invasive option, using a diode laser probe under endoscopic guidance to create a fistulous tract [4]. TL-DCR competes favourably on operative time and cosmesis, though its long-term efficacy remains a matter of ongoing debate [5].

Traditionally, success in lacrimal surgery has been defined by anatomical patency on irrigation or imaging. However, anatomical success does not always fully reflect patient-perceived symptomatic improvement or postoperative QoL, highlighting the importance of evaluating patient-centered outcomes alongside objective surgical measures [6]. Consequently, standardized patient-reported outcome measures (PROMs) have gained increasing importance as tools for assessing surgical effectiveness from the patient’s perspective [7]. Several PROM instruments have been used in lacrimal drainage disorders and DCR surgery, including generic health-related QoL questionnaires such as the Short Form-36 and the Glasgow Benefit Inventory (GBI), as well as disease-specific tools designed to better assess epiphora-related symptom burden and psychosocial impairment [8].

The GBI is a validated, procedure independent questionnaire widely used across surgical specialties to assess patients’ perceived benefit following intervention [9,10]. However, its generic design may limit its sensitivity to the specific impact of lacrimal disease. The Lacrimal Symptom Questionnaire (Lac-Q) was developed to address precisely this gap, quantifying both symptom burden and psychosocial consequences in patients with lacrimal drainage disorders [11]. In the present study, we selected Lac-Q because of its validated disease-specific structure and combined it with the GBI to obtain both targeted and global assessments of postoperative patient-reported outcomes.

This prospective study aimed to compare the effects of EX-DCR, EN-DCR, and TL-DCR on patient-reported QoL in individuals undergoing surgery for NLDO. Functional improvement, symptom relief, and overall satisfaction were evaluated using the Lac-Q questionnaire and GBI, alongside the Munk score. By combining objective and subjective outcome measures, the study aims to provide a more comprehensive comparison of contemporary DCR techniques from a patient-centered perspective.

## 2. Methods

This prospective study was conducted at the Department of Ophthalmology of a tertiary care training and research hospital between October 2021 and December 2022 and included 69 patients diagnosed with primary NLDO who presented with epiphora. Institutional ethical committee approval was obtained prior to the commencement of the study. All researchers adhered to the principles of the Declaration of Helsinki, and written informed consent was obtained from all participants.

All patients included in the study underwent a comprehensive evaluation to confirm the diagnosis of NLDO which was defined as symptomatic distal lacrimal drainage obstruction characterized by persistent epiphora and confirmed by nasolacrimal irrigation findings demonstrating impaired passage into the nasal cavity. This evaluation included detailed history taking (covering the onset of epiphora, history of acute dacryocystitis, prior surgeries, family history, systemic diseases, medications, and bleeding diathesis), a complete ophthalmological examination, and nasolacrimal irrigation. Preoperative CT imaging was requested only for patients with a history of trauma, a suspected mass, or potential anatomical variations (e.g., concha bullosa). All patients underwent a detailed nasal endoscopic examination using a Karl-Storz^®^ (Karl Storz SE & Co. KG, Tuttlingen, Germany) endoscopy system, which included a 250-watt fiberoptic light source and 4 mm rigid endoscopes with 0° and 30° angles. The exclusion criteria included epiphora caused by conditions other than NLDO, obstruction of the upper lacrimal system (punctum or canaliculus), active infections of the nasal or lacrimal system, and nasal pathologies severe enough to preclude surgery (e.g., septal deviation or turbinate hypertrophy). Following ophthalmologic and otorhinolaryngologic evaluation, patients were informed about the advantages and disadvantages of each surgical technique. Surgical selection was primarily based on patient preference; however, nasal anatomical factors identified during preoperative endoscopic examination, including significant septal deviation, narrow nasal cavity, and other intranasal abnormalities, were also considered when determining the suitability of endonasal approaches. The total duration of the operation, along with postoperative pain scores from the first night and the first day, were recorded for all patients.

### 2.1. Subjective Indicators of Well-Being, Patient Satisfaction and Improvement in QoL

#### 2.1.1. Munk Score

The Munk score, a subjective grading system ranging from 0 (no tearing) to 5 (constant and severe tearing with visible overflow onto the face), was utilized to assess the severity of epiphora preoperatively and at the sixth postoperative month (Supplementary Table S1).

#### 2.1.2. Lacrimal Symptom (Lac-Q) Questionnaire

The Lac-Q questionnaire, a patient-reported outcome measure, was administered to patients both preoperatively and at each postoperative follow-up visit to evaluate surgical outcomes and assess improvements in symptoms and QoL. The Lac-Q consists of two parts: the first includes five questions to assess the social impact of the disease (with a range from 0 = no negative impact to 5 = maximum negative impact), and the second evaluates four different lacrimal symptoms associated with NLDO (Supplementary Figure S1).

#### 2.1.3. The Glasgow Benefit Inventory (GBI)

GBI, a widely used questionnaire [10] designed to assess the impact of surgical procedures in a patient’s daily lives, was administered at each postoperative visit. It consists of several questions that measure general well-being, social, and physical health. Responses are scored on a five-point Likert scale, and the total score ranges from −100 (indicating maximum harm) to 0 (no benefit/change) and +100 (indicating maximum benefit) (Supplementary Table S2).

### 2.2. Surgical Techniques

All surgeries were performed under general anesthesia. Preoperatively, nasal decongestion was achieved using oxymetazoline spray and nasal tampons soaked in an adrenaline-lidocaine solution.

For the EX-DCR technique, a 15–20 mm skin incision was made parallel to the nasal bridge, 8–10 mm from the medial canthal ligament, taking care to preserve deep tissues and avoid trauma to the angular vein. After exposing the lacrimal bone through dissection, a 12 × 14 mm bony window was created using a Kerrison rongeur. A horizontal H-shaped incision was made in the lacrimal sac and nasal mucosa, which were then sutured with 6/0 absorbable polyglactin suture to form opposing anterior flaps. A bicanalicular silicone tube was placed, and the wound was closed in layers.

For the EN-DCR technique, a 4 mm 0° nasal endoscope and Karl Storz^®^ HD imaging system (Karl Storz SE & Co. KG, Tuttlingen, Germany) were used. A transcanalicular laser probe was advanced through the upper canaliculus to confirm localization via illumination. Following a nasal mucosal incision, a bony ostium was created and enlarged using Smith-Kerrison forceps under endoscopic visualization. No powered drilling system was used. Adequate exposure of the lacrimal sac fundus was achieved through enlargement of the bony ostium and careful removal of the surrounding lacrimal bone. The medial (anterior) wall of the lacrimal sac was then incised and excised to create an approximately 15 × 15 mm rhinostomy opening, followed by bicanalicular silicone tube intubation.

For TL-DCR technique, after dilating the lacrimal puncta, a 980 nm 600 µm diode laser probe (D-plus Qvanta-System^®^ Multidiod Laser, QVANTA System S.p.A., Samarate, Italy) was advanced through the lower canaliculus to the lacrimal sac, guided by endoscopic visualization. A fistula of approximately 8 × 12 mm was created between the lacrimal sac and nasal cavity using 10 watt laser pulses, followed by bicanalicular silicone tube intubation. The procedure concluded with irrigation and careful aspiration to protect surrounding tissues.

Postoperatively, antibiotic eye drops (topical moxifloxacin, 4× daily) and steroid eye drops (topical dexamethasone, 4× daily) were prescribed. Additionally, to prevent secondary infections, oral amoxicillin-clavulanic acid (1000 mg, twice daily) was administered for one week, along with oral analgesics and anti-inflammatory medications. Nasal saline irrigations were also recommended postoperatively to promote mucosal healing and reduce crust formation. Patients were followed up on the first day, first week, first month, third month, and subsequently at three-month intervals after the operation. Postoperative anatomical patency was primarily evaluated by lacrimal irrigation together with assessment of symptomatic improvement. The Lac-Q and GBI questionnaires were administered in a paper-based format during outpatient follow-up visits by the study investigators. Lac-Q assessments were performed preoperatively and at each postoperative follow-up, whereas GBI was administered at each postoperative visit. All patients included in the final analysis completed the questionnaires. Routine standardized endonasal evaluation of the ostium, fluorescein dye flow, or intranasal adhesion formation was not systematically performed in all patients. Anatomical success was defined as patency on postoperative lacrimal irrigation, while functional success was defined as a postoperative Munk score ≤1, indicating no epiphora or only occasional tearing requiring dabbing less than twice daily. All procedures were performed by one oculoplastic surgeons (HF) with >15 years of experience in lacrimal surgery and >100 cases in each technique.

### 2.3. Statistical Analysis

Statistical analyses were performed using IBM SPSS Statistics for Windows, version 29.0 (IBM Corp., Armonk, NY, USA). Sample size calculation was conducted using G*Power software (v3.1.9.7, Heinrich-Heine-Universität Düsseldorf, Germany). Based on an anticipated effect size of 0.4, a significance level of 0.05, and a statistical power of 80%, a minimum of 60 patients (at least 20 per group) was required. The final sample size of 69 patients met this requirement. The distribution of continuous variables was assessed using visual (histograms, probability plots) and analytical methods (Shapiro–Wilk test). Continuous variables were expressed as mean ± standard deviation or median (minimum–maximum), as appropriate, while categorical variables were presented as frequencies and percentages. Comparisons of continuous variables among the three groups were performed using one-way analysis of variance (ANOVA), with Bonferroni post hoc tests applied for pairwise comparisons when appropriate. Homogeneity of variances was assessed using Levene’s test. Categorical variables were compared using the chi-square test or Fisher’s exact test, as appropriate. Changes over time in repeated measurements (postoperative pain scores, Munk scores, Lac-Q scores, and GBI scores) were analyzed using repeated measures ANOVA, including assessment of group × time interaction effects. A *p*-value of <0.05 was considered statistically significant.

## 3. Results

A total of 69 patients diagnosed with primary NLDO were included in this study. Of these, 45 (65%) were female and 24 (34.8%) were male. The mean age of the patients was 58.99 ± 14.86 years (range: 26–88 years), and the mean follow-up period was 17.19 ± 4.07 months (range: 6–26 months). The distribution of surgical techniques was 25 (36.2%) EX-DCR, 21 (30.4%) EN-DCR, and 23 (33.3%) TL-DCR. The mean duration of bicanalicular silicone tube placement was 4.97 ± 1.04 months (range: 2–6.5 months) (Table 1).

There was no statistically significant difference between the groups in terms of age, gender, side of NLDO (right vs. left), duration of symptoms, or presence of additional systemic conditions (such as diabetes mellitus, hypertension, or rheumatological diseases) across the surgical techniques. The EX-DCR group had a significantly longer follow-up period compared to both EN-DCR and TL-DCR groups, while no significant difference was observed between EN-DCR and TL-DCR. The longer follow-up duration in the EX-DCR group primarily reflected the earlier implementation and more frequent use of this technique during the initial phase of patient recruitment. A significant difference in operation time was observed among all three techniques, with EX-DCR having the longest duration, followed by EN-DCR, and TL-DCR demonstrating the shortest operative time (*p* < 0.001) (Table 1).

### 3.1. Preoperative and Postoperative Assessment

Pre- and postoperative Munk scores are presented in Table 2. Preoperative Munk scores did not differ significantly among the three groups (*p* > 0.05), suggesting comparable baseline symptom severity. The mean Munk score, reflecting the severity of epiphora, decreased significantly in all groups at the 6th postoperative month compared with the preoperative period (*p* ≤ 0.001), indicating marked functional improvement. No significant difference was observed in the amount of improvement among the three surgical techniques (Group × Time interaction, *p* = 0.159).

### 3.2. Pain Scores

Pain was assessed on the first night and the first morning after surgery. Repeated measures ANOVA showed a significant reduction in pain over time within all groups (*p* < 0.001), whereas the trajectory of pain reduction did not differ significantly between surgical techniques (Group × Time interaction, *p* = 0.502) (Table 3).

### 3.3. Anatomical Success

Anatomical success was defined as postoperative patency of the nasolacrimal system on irrigation. The anatomical success rates were 92% for EX-DCR, 90.5% for EN-DCR, and 91.3% for TL-DCR. No statistically significant difference was observed between the surgical techniques (*p* = 0.867) (Table 4).

### 3.4. Functional Success

Functional success was defined as a reduction in epiphora symptoms, indicated by a Munk score of ≤1. The functional success rates were 88% for EX-DCR, 90.5% for EN-DCR, and 82.7% for TL-DCR. No statistically significant difference in functional success was observed among the surgical techniques (*p* = 0.429) (Table 4).

### 3.5. Quality of Life

The Lacrimal Symptom Questionnaire (Lac-Q), which evaluates the impact of NLDO on lacrimal symptoms, showed no statistically significant differences between the three groups at any time point, including preoperative and postoperative assessments (*p* > 0.05) (Table 5).

**Figure 1:**
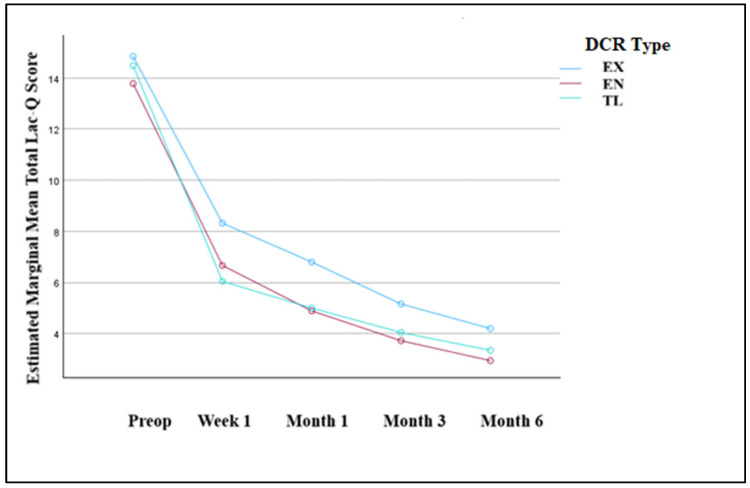
Changes in estimated marginal mean total Lac-Q scores from baseline to postoperative month 6 according to DCR technique (EX-DCR, EN-DCR, and TL-DCR).

A significant improvement in Lac-Q scores over time was observed in all groups (*p* < 0.001). This indicates a progressive reduction in symptom severity following surgery, regardless of the surgical technique used. However, no significant difference was observed in the pattern of improvement among the three groups (Group × Time interaction, *p* = 0.798) (Figure 1).

The Glasgow Benefit Inventory (GBI) was used to evaluate postoperative changes in quality of life. No statistically significant differences in GBI scores were observed between the three groups at any postoperative time point (*p* > 0.05) (Table 6).

**Figure 2:**
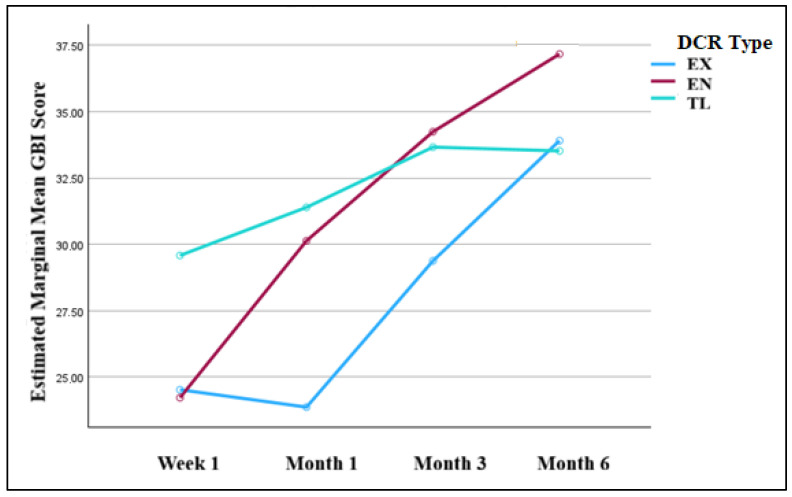
Changes in estimated marginal mean Glasgow Benefit Inventory (GBI) scores from postoperative week 1 to postoperative month 6 according to DCR technique (EX-DCR, EN-DCR, and TL-DCR).

Analysis of changes over time demonstrated a statistically significant improvement in GBI scores in the EX-DCR (*p* = 0.019) and EN-DCR groups (*p* < 0.001), whereas no significant change was observed in the TL-DCR group (*p* = 0.171). However, the pattern of change over time did not differ significantly among the three techniques (Group × Time interaction, *p* = 0.210) (Figure 2).

No intraoperative complications, including periorbital edema or ecchymosis, angular vein injury, corneal erosion, conjunctivitis, or punctal damage, were observed in any of the surgical groups. Similarly, no major postoperative complications, such as pseudoepicanthal fold, keloid formation, severe epistaxis, intranasal adhesion, wound infection, or fibrosis, were detected. Two silicone tube–related complications were recorded. In the EN-DCR group, one patient developed punctal stenosis at the first postoperative month, requiring surgical revision. In the EX-DCR group, one patient experienced cheese-wiring of the silicone tube at the third postoperative month, necessitating tube removal.

Patients with persistent epiphora or restenosis were managed individually according to clinical findings, including revision surgery, repeat silicone tube intubation, or close follow-up where appropriate.

## 4. Discussion

In this prospective comparative study, EX-DCR, EN-DCR, and TL-DCR demonstrated comparable anatomical and functional outcomes in patients with primary NLDO. Significant postoperative improvements were observed in Munk, Lac-Q, and GBI scores across all groups, emphasizing the beneficial impact of DCR surgery on patient-reported symptoms and QoL. Although EN-DCR demonstrated numerically greater improvements in some QoL measures, these differences did not reach statistical significance.

EX-DCR remains the reference standard for the surgical treatment of NLDO because of its high anatomical and functional success rates [12]. Consistent with previous reports [2], EX-DCR achieved the numerically highest anatomical success rate (92%) in our cohort, with a functional success rate of 88%, although the difference was not statistically significant.

EN-DCR demonstrated anatomical and functional success rates comparable to those of EX-DCR, in agreement with previous studies [13,14,15]. Although postoperative Lac-Q and GBI scores did not differ significantly among groups, numerically greater improvements were observed in the EN-DCR group throughout follow-up. However, these differences did not reach statistical significance and should be interpreted cautiously.

TL-DCR has been introduced as a minimally invasive alternative to conventional DCR techniques, although its long-term efficacy remains controversial. In a recent network meta-analysis including 3277 cases, Eveklioğlu et al. reported lower success rates for TL-DCR than for both EX-DCR and EN-DCR [16]. Similarly, several studies have reported anatomical success rates ranging from approximately 34% to 95%, highlighting substantial variability in long-term outcomes [17]. In the present study, TL-DCR achieved an anatomical success rate of 91.3% and a functional success rate of 82.7%. These outcomes may have been influenced by factors such as surgeon experience, standardized bicanalicular silicone intubation, careful patient selection, and the relatively shorter follow-up duration in the TL-DCR group. However, the limited sample size and variability of long-term outcomes reported in the literature should be considered when interpreting these findings. Furthermore, thermal injury and limitations in fiberoptic maneuverability may contribute to proximal lacrimal system stenosis and surgical failure; notably, proximal stenosis was identified in two anatomically unsuccessful TL-DCR cases in our series.

Functional outcomes were comparable among the three techniques, with EN-DCR demonstrating the numerically highest functional success rate (90.5%), although no statistically significant differences were detected between groups. The modest discrepancies between anatomical and functional outcomes observed across techniques may reflect factors beyond anatomical patency alone, including lacrimal pump function and ostium characteristics. However, these potential mechanisms were not specifically evaluated in the present study.

An important strength of the present study is the prospective evaluation of PROMs using both the disease-specific Lac-Q questionnaire and the generic QoL instrument GBI. Recent revalidation studies have further strengthened the role of Lac-Q as a reliable and standardized disease-specific PROM for assessing both symptom severity and QoL impairment in patients with NLDO [18]. Although anatomical patency has traditionally been considered the primary indicator of surgical success in lacrimal surgery, increasing evidence suggests that objective patency alone may not fully reflect postoperative patient satisfaction or symptomatic improvement [19]. In our cohort, all three DCR techniques were associated with marked improvements in both Lac-Q and GBI scores, indicating that successful restoration of lacrimal drainage substantially improves symptom burden, social functioning, and overall patient well-being. The use of both instruments provided complementary perspectives on surgical outcomes, with Lac-Q evaluating epiphora-related symptoms and psychosocial impact, and GBI assessing broader QoL benefits following surgery.

Although no statistically significant intergroup differences were observed in either GBI or Lac-Q outcomes, the EN-DCR group demonstrated numerically greater improvements throughout follow-up. However, these differences did not reach statistical significance and should be interpreted cautiously. Previous studies evaluating PROMs after DCR surgery have similarly reported significant postoperative improvements in patient-reported symptoms and QoL. Sipkova et al. used the Lac-Q questionnaire together with the Glasgow Benefit Inventory and demonstrated substantial postoperative improvements in both symptom burden and quality of life following surgery for epiphora, emphasizing the importance of patient-centered outcome assessment alongside anatomical success measures [7]. Similarly, in a study evaluating powered EN-DCR using the Lac-Q questionnaire, significant improvements were observed in both symptom and social impact scores, with postoperative Lac-Q scores correlating closely with anatomical and functional success rates. The authors concluded that Lac-Q was sensitive to clinical improvement and effectively reflected changes in patient-reported outcomes following surgery [20]. More recently, Dave et al. reported comparable functional and QoL outcomes between endonasal and external DCR despite differences in surgical approach [6]. Yeniad et al. have noted comparable QoL outcomes between EX-DCR and TL-DCR despite differences in anatomical success [21]. Collectively, these studies and our findings support the incorporation of PROMs alongside conventional anatomical and functional outcome measures in the evaluation of lacrimal surgery outcomes.

Several limitations of this study merit consideration. Patients were not randomized across surgical techniques; the choice of procedure (EX-DCR, EN-DCR, or TL-DCR) was influenced by patient preference and nasal anatomical findings. This non-randomized allocation may have introduced selection bias and affected not only postoperative QoL outcomes, but also anatomical and functional success rates, operative times, postoperative pain scores, and recovery characteristics. In addition, subjective outcome measures may have been influenced by differences in patient expectations regarding minimally invasive approaches. Postoperative anatomical success was evaluated using lacrimal irrigation. Routine standardized endonasal examination of the ostium, fluorescein flow, or intranasal adhesions was not systematically performed in all patients. The absence of these additional objective endonasal evaluations may have limited detailed assessment of ostium healing and restenosis mechanisms. Follow-up duration was not uniform among the three surgical groups. Patients undergoing EX-DCR had significantly longer follow-up periods compared with the EN-DCR and TL-DCR groups. Because restenosis and recurrent epiphora may develop months or even years after surgery, the shorter follow-up in the EN-DCR and TL-DCR groups may have resulted in underestimation of late anatomical or functional failure in these techniques [22,23]. In addition, the relatively small number of unsuccessful or reoperated cases limited the ability to perform reliable subgroup analyses of postoperative Lac-Q outcomes in these patients. The preoperative duration of epiphora symptoms was not systematically recorded. Symptom duration may represent a potential confounding factor and could have influenced surgical outcomes. Additionally, although sample size calculations met the predefined statistical assumptions, the relatively limited sample size in this three-group comparison may have increased the risk of type II error and reduced the ability to detect small differences between surgical techniques. Future prospective studies with larger cohorts and standardized long-term follow-up are needed to better define the relationship between anatomical success, functional outcomes, and patient-reported QoL following different DCR techniques.

In conclusion, all three DCR techniques evaluated in this study demonstrated high anatomical and functional success rates, with no statistically significant differences observed between them. Although EX-DCR showed the numerically highest anatomical success rate, EN-DCR was associated with shorter operative time and numerically greater improvements in PROMs. TL-DCR provided comparable anatomical results, although functional success rates and PROM improvements were numerically lower than those observed in the other groups. These findings suggest that EX-DCR, EN-DCR, and TL-DCR are all effective treatment options for NLDO, and that the choice of surgical approach should be individualized according to patient characteristics, anatomical considerations, and surgeon experience.

## Figures and Tables

**Table 1:** Comparison of Demographic and Surgical Parameters Across DCR Techniques.

Parameters	DCR Technique	n	Mean ± SD	Min-Max (Median)	^a^ *p*-Value (Overall)	*p* (1–2)	*p* (1–3)	*p* (2–3)
Age (years)	EX (1)	25	60.08 ± 13.56	34–80 (58)	0.728	1.000	1.000	1.000
	EN (2)	21	57.48 ± 12.64	30–80 (58)				
	TL (3)	23	59.17 ± 18.25	26–88 (62)				
Follow-up time (months)	EX (1)	25	20.00 ± 3.84	10–26 (20)	<0.001	0.003	<0.001	0.619
	EN (2)	21	16.43 ± 3.44	6–24 (17)				
	TL (3)	23	14.83 ± 3.00	10–21 (15)				
Stent duration time (months)	EX (1)	25	5.08 ± 1.00	3–6.5 (5)	0.388	0.816	1.000	0.908
	EN (2)	21	4.74 ± 1.03	2–6 (5)				
	TL (3)	23	5.07 ± 1.10	2–6 (5.5)				
Total duration of operation (min)	EX (1)	25	94.40 ± 16.35	65–120 (95)	<0.001	<0.001	<0.001	0.006
	EN (2)	21	55.24 ± 7.16	45–75 (55)				
	TL (3)	23	44.13 ± 7.49	35–70 (45)				

EX (1): External; EN (2): Endonasal; TL (3): Transcanalicular Laser. SD: Standard Deviation; Min: Minimum; Max: Maximum. ^a^ *p*-value (Overall): Calculated using One-Way ANOVA for the overall comparison among the three groups. *p* (1–2), *p* (1–3), *p* (2–3): Indicate pairwise comparisons calculated using the Bonferroni post hoc test, as the assumption of homogeneity of variances was met.

**Table 2:** Comparison of Preoperative and Postoperative 6th Month Munk Scores (Mean ± SD), (Functional Success) Among DCR Types.

DCR Type	Preop Munk Score	Postop 6th Month Munk Score	^a^ *p*-Value (Time)	^a^ *p*-Value (Group × Time)
EX	3.80 ± 0.41	0.68 ± 0.90	0.001	0.159
EN	4.00 ± 0.00	0.42 ± 0.77	<0.001	
TL	3.78 ± 0.42	0.68 ± 0.78	0.001	

DCR: Dacryocystorhinostomy; EX: External; EN: Endonasal; TL: Transcanalicular Laser. Preop: Preoperative; Postop: Postoperative; SD: Standard Deviation. ^a^ *p*-value (Time): Indicates the significance of the reduction in Munk scores from the preoperative period to the postoperative 6th month within each group (Repeated measures ANOVA). ^a^ *p*-value (Group × Time): Indicates the interaction effect between the surgical group and time, demonstrating no significant difference in the amount of functional improvement among the three groups (Repeated measures ANOVA).

**Table 3:** Comparison of Postoperative Pain Scores (Mean ± SD) [Median (Min–Max)] Among DCR Types.

DCR Type	Postop First Night Pain	Postop First Morning Pain	^a^ *p*-Value (Time)	^a^ *p*-Value (Group × Time)
EX	4.08 ± 1.78 [4.00 (1.00–7.00)]	2.64 ± 1.58 [2.00 (0.00–6.00)]	<0.001	0.502
EN	3.57 ± 1.33 [3.00 (1.00–6.00)]	1.81 ± 0.87 [2.00 (1.00–4.00)]	<0.001	
TL	3.39 ± 0.89 [3.00 (2.00–5.00)]	1.61 ± 0.94 [2.00 (0.00–4.00)]	<0.001	

DCR: Dacryocystorhinostomy; EX: External; EN: Endonasal; TL: Transcanalicular Laser. Postop: Postoperative; SD: Standard Deviation; Min: Minimum; Max: Maximum. Pain scores were evaluated postoperatively at the first night and the first morning. ^a^ *p*-value (Time): Indicates the significance of the reduction in pain scores from the 1st night to the 1st morning within each surgical group (Repeated measures ANOVA). ^a^ *p*-value (Group × Time): Indicates the interaction effect between the surgical group and time, demonstrating no significant difference in the trajectory of pain reduction among the three groups (Repeated measures ANOVA).

**Table 4:** Anatomical and Functional Success Rates Based on the Applied DCR Technique.

Parameters	EX	EN	TL	^a^ *p*-Value
Anatomical Success (%)	92.0	90.5	91.3	0.867
Functional Success (%)	88.0	90.5	82.7	0.429

DCR: Dacryocystorhinostomy. EX: External; EN: Endonasal; TL: Transcanalicular Laser. ^a^ *p*-value: Calculated using the Chi-square test (or Fisher’s exact test). *p*-value > 0.05 indicates that there is no statistically significant difference among the three surgical techniques regarding both anatomical and functional success.

**Table 5:** Comparison of Preoperative and Postoperative Total Lac-Q Scores (Mean ± SD) Among DCR Types Over Time.

DCR Type	Preop	Postop 1st Week	Postop 1st Month	Postop 3rd Month	Postop 6th Month	^a^ *p*-Value (Time)	^a^ *p*-Value (Group × Time)
EX	14.84 ± 4.44	8.32 ± 4.28	6.80 ± 4.34	5.16 ± 4.49	4.20 ± 4.13	<0.001	0.798
EN	14.24 ± 3.35	6.57 ± 2.96	4.95 ± 2.69	4.00 ± 2.60	2.84 ± 1.80	<0.001	
TL	14.48 ± 5.05	6.04 ± 4.24	5.00 ± 3.02	4.04 ± 2.53	3.35 ± 2.19	<0.001	

DCR: Dacryocystorhinostomy; EX: External; EN: Endonasal; TL: Trans canalicular Laser. Lac-Q: Lacrimal Symptom Questionnaire; Preop: Preoperative; Postop: Postoperative; SD: Standard Deviation. ^a^ *p*-value (Time): Indicates the significance of the change in Lac-Q scores over time within each group (Repeated measures ANOVA). ^a^ *p*-value (Group × Time): Indicates the interaction effect between the surgical group and time, showing no significant difference in the improvement trends among the three groups (Repeated measures ANOVA).

**Table 6:** Comparison of Postoperative Glasgow Benefit Inventory (GBI) Scores (Mean ± SD) Across Different DCR Techniques Over Time.

DCR Type	Postop 1st Week	Postop 1st Month	Postop 3rd Month	Postop 6th Month	^a^ *p*-Value (Time)	^a^ *p*-Value (Group × Time)
EX	24.53 ± 16.12	23.87 ± 14.96	29.38 ± 16.38	33.91 ± 17.47	0.019	0.210
EN	24.26 ± 8.75	30.34 ± 10.28	34.25 ± 10.66	37.17 ± 11.63	<0.001	
TL	29.58 ± 12.58	31.40 ± 12.82	33.67 ± 13.11	33.52 ± 13.15	0.171	

DCR: Dacryocystorhinostomy; EX: External; EN: Endonasal; TL: Transcanalicular Laser. GBI: Glasgow Benefit Inventory; Postop: Postoperative; SD: Standard Deviation. ^a^ *p*-value (Time): Indicates the significance of the change in total GBI scores over time within each group (Repeated measures ANOVA). Note that the change is not significant for the transcanalicular laser group. ^a^ *p*-value (Group × Time): Indicates the interaction effect between the surgical group and time, showing no significant difference in the overall trend of scores among the three groups (Repeated measures ANOVA).

## Data Availability

The original contributions presented in this study are included in the article, further inquiries can be directed to the corresponding author.

## Supplementary Materials

The following supporting information can be downloaded at: https://www.mdpi.com/article/10.3390/jcm15124488/s1, Table S1. The Munk scoring system. Figure S1. The Lacrimal Symptom Questionnaire. Table S2. The Glasgow Benefit Inventory (GBI).
