# Supplementary material for: Comparison of the Impact of Different Dacryocystorhinostomy Techniques on Patient Quality of Life in Lacrimal Duct Obstruction
Source: J Clin Med. 2026 Jun 10;15(12):4488. doi: 10.3390/jcm15124488 (PMC13301723; doi:10.3390/jcm15124488)
Supplement: Supplementary file 1 [file jcm-15-04488-s001.zip › jcm-4273533-supplementary.pdf]

Supplementary Materials

Table S1. The Munk scoring system.

GRADE	MUNK SCALE
**0**	No epiphora
**1**	Epiphora requiring dabbing less than twice a day
**2**	Epiphora requiring dabbing 2–4 times a day
**3**	Epiphora requiring dabbing 5–10 times a day
**4**	Epiphora requiring dabbing more than 10 times a day
**5**	Constant epiphora

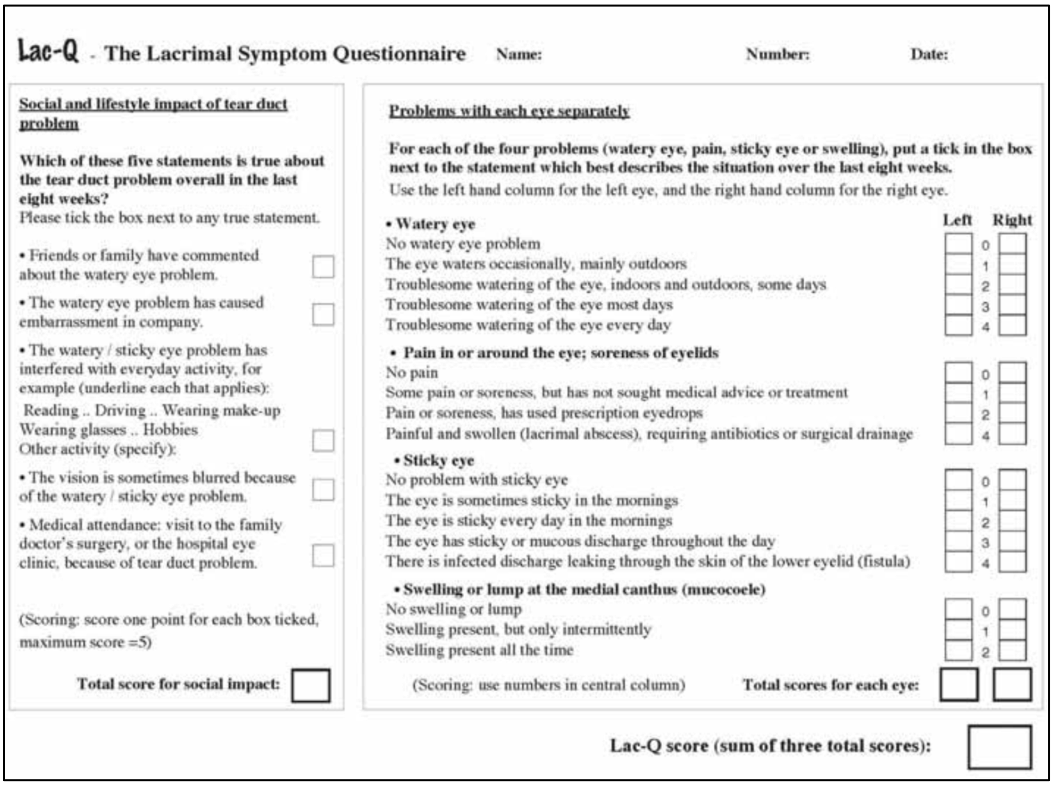

Lac-Q - **The Lacrimal Symptom Questionnaire** **Name:** **Number:** **Date:**

Social and lifestyle impact of tear duct problem

Which of these five statements is true about the tear duct problem overall in the last eight weeks?
Please tick the box next to any true statement.

- Friends or family have commented about the watery eye problem. ☐
- The watery eye problem has caused embarrassment in company. ☐
- The watery / sticky eye problem has interfered with everyday activity, for example (underline each that applies): Reading .. Driving .. Wearing make-up Wearing glasses .. Hobbies Other activity (specify): ☐
- The vision is sometimes blurred because of the watery / sticky eye problem. ☐
- Medical attendance: visit to the family doctor's surgery, or the hospital eye clinic, because of tear duct problem. ☐

(Scoring: score one point for each box ticked, maximum score =5)

Total score for social impact: ☐

Problems with each eye separately

For each of the four problems (watery eye, pain, sticky eye or swelling), put a tick in the box next to the statement which best describes the situation over the last eight weeks.
Use the left hand column for the left eye, and the right hand column for the right eye.

	Left		Right
• **Watery eye**			
No watery eye problem	☐	0	☐
The eye waters occasionally, mainly outdoors	☐	1	☐
Troublesome watering of the eye, indoors and outdoors, some days	☐	2	☐
Troublesome watering of the eye most days	☐	3	☐
Troublesome watering of the eye every day	☐	4	☐
• **Pain in or around the eye; soreness of eyelids**			
No pain	☐	0	☐
Some pain or soreness, but has not sought medical advice or treatment	☐	1	☐
Pain or soreness, has used prescription eyedrops	☐	2	☐
Painful and swollen (lacrimal abscess), requiring antibiotics or surgical drainage	☐	4	☐
• **Sticky eye**			
No problem with sticky eye	☐	0	☐
The eye is sometimes sticky in the mornings	☐	1	☐
The eye is sticky every day in the mornings	☐	2	☐
The eye has sticky or mucous discharge throughout the day	☐	3	☐
There is infected discharge leaking through the skin of the lower eyelid (fistula)	☐	4	☐
• **Swelling or lump at the medial canthus (mucocoele)**			
No swelling or lump	☐	0	☐
Swelling present, but only intermittently	☐	1	☐
Swelling present all the time	☐	2	☐
(Scoring: use numbers in central column) **Total scores for each eye:**	☐		☐

Lac-Q score (sum of three total scores): ☐

Figure S1. The Lacrimal Symptom Questionnaire.

Table S2. The Glasgow Benefit Inventory (GBI).

How to Complete This Questionnaire:

This questionnaire is designed to find out what sort of effects your recent operation has had on your lifestyle, general well-being, etc.
Some of the effects below may apply to you, some may not.
Please answer **all** questions by selecting the number that best reflects how your epiphora has affected you **since your operation**
Please Note—some of the questions are scored in **reverse order** (i.e., 5 to 1, rather than 1 to 5)**,** so please read the statement and the scores carefully

1 = Much **worse**
2 = A little or somewhat **worse**
3 = No change
4 = A little or somewhat **better**
5 = Much **better**

1. Has the result of the operation affected the things you do?	1 2 3 4 5
2. Have the results of the operation made your overall life better or worse?	5 4 3 2 1
3. Since the operation, have you felt more or less optimistic about the future?	5 4 3 2 1
4. Since your operation, do you feel more or less embarrassed when with a group of people?	1 2 3 4 5
5. Since your operation, do you have more or less self-confidence?	5 4 3 2 1
6. Since your operation, have you found it easier or harder to deal with company?	5 4 3 2 1

7. Since your operation, do you feel that you have more or less support from your friends?	5 4 3 2 1
8. Have you been to your family doctor, for any reason, more or less often, since your operation?	1 2 3 4 5
9. Since your operation, do you feel more or less confident about job opportunities?	5 4 3 2 1
10. Since your operation, do you feel more or less self-conscious?	1 2 3 4 5
11. Since your operation, are the more or fewer people who really care about you?	5 4 3 2 1
12. Since you had the operation, do you catch colds or infections more or less often?	1 2 3 4 5
13. Have you had to take more or less medicine for any reason, since your operation?	1 2 3 4 5
14. Since your operation, do you feel better or worse about yourself?	5 4 3 2 1
15. Since your operation, do you feel that you have had more or less support from your family?	5 4 3 2 1
16. Since your operation, are you more or less inconvenienced by your health problem?	1 2 3 4 5
17. Since your operation, have you been able to participate in more or fewer social activities?	5 4 3 2 1
18. Since your operation, have you been more or less inclined to withdraw from social situations?	1 2 3 4 5
TOTAL = [(sum responses / 18) − 3] × 50	

https://doi.org/10.3390/jcm15124488
